# (*E*)*-N*,*N*-Diethyl-2,6-diisopropyl-4-[2-(4-nitro­phen­yl)ethen­yl]aniline

**DOI:** 10.1107/S1600536813030948

**Published:** 2013-11-20

**Authors:** Christoph Wink, Dieter Schollmeyer, Heiner Detert

**Affiliations:** a University Mainz, Duesbergweg 10-14, 55099 Mainz, Germany

## Abstract

The title compound, C_24_H_32_N_2_O_2_, was prepared by Horner olefination of 4-di­ethyl­amino-3,5-diiso­propyl­benzaldehyde and diethyl *p*-nitro­benzyl­phospho­nate. There are two independent mol­ecules (*A* and *B*) in the asymmetric unit. Their main axes, defined by the line connecting the N atoms of the nitro and amino groups, open an angle of 79.42 (3)°. Steric hindrance around the amino group is reflected in a long aryl C—N bond [1.434 (3) Å for mol­ecule *A* and 1.440 (3) Å for mol­ecule *B*], a pyramidal geometry [angle sum = 350.0 (2)° for mol­ecule *A* and 349.6 (2)° for mol­ecule *B*], and dihedral angles between the phenyl­ene group and the plane defined by the CH_2_—N—CH_2_ unit of 86.9 (3)° for mol­ecule *A* and 88.3 (3)° for mol­ecule *B*. This gives structural support for the electronic decoupling of the amino group from the nearly planar nitro­stilbene moiety (r.m.s. deviation for C, N and O atoms = 0.097 for mol­ecule *A* and 0.107 Å for mol­ecule *B*).

## Related literature

For the synthesis of amino­nitro­stilbenes, see: Pfeiffer *et al.* (1915 ▶); Meier *et al.* (2004 ▶). For torsion-depent optical properties, see: Baumann *et al.* (1977 ▶); Wink & Detert (2013 ▶); Dekhtyar & Rettig (2007 ▶). For conjugated oligomers as sensing materials, see: Schmitt *et al.* (2008 ▶); Zucchero *et al.* (2009 ▶). For the structures of donor–acceptor stilbenoid dyes, see: Schollmeyer & Detert (2011 ▶); Fischer *et al.* (2011 ▶). For structures of sterically crowded push–pull analogues, see: Wink & Detert (2013 ▶). For the synthesis of the starting materiel, see: Wink *et al.* (2011 ▶). For chromophores and fluoro­phores based on quadrupolar donor–acceptor-substituted stilbenoid systems, see: Detert & Sugiono (2005 ▶); Schmitt *et al.* (2013 ▶); Nemkovich *et al.* (2010 ▶).
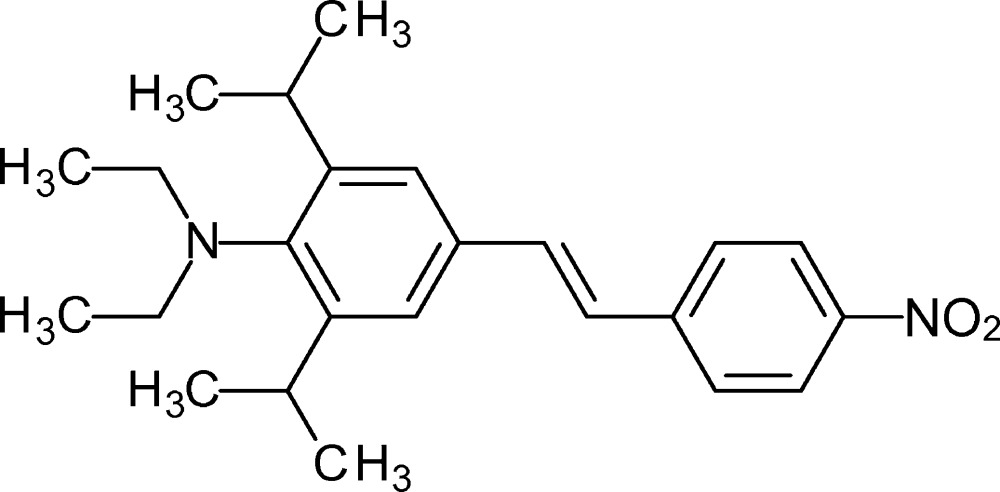


## Experimental

### 

#### Crystal data

C_24_H_32_N_2_O_2_
*M* *_r_* = 380.52
Triclinic, 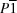

*a* = 7.3477 (7) Å
*b* = 15.4143 (14) Å
*c* = 20.3747 (19) Å
α = 104.642 (8)°
β = 92.414 (8)°
γ = 90.793 (7)°
*V* = 2230.0 (4) Å^3^
*Z* = 4
Mo *K*α radiation
μ = 0.07 mm^−1^
*T* = 193 K
0.60 × 0.10 × 0.05 mm

#### Data collection

Stoe IPDS 2T diffractometer
21656 measured reflections
10640 independent reflections
3887 reflections with *I* > 2σ(*I*)
*R*_int_ = 0.086

#### Refinement

*R*[*F* ^2^ > 2σ(*F* ^2^)] = 0.057
*wR*(*F* ^2^) = 0.174
*S* = 0.79
10640 reflections
518 parameters
H-atom parameters constrained
Δρ_max_ = 0.25 e Å^−3^
Δρ_min_ = −0.24 e Å^−3^

### 

Data collection: *X-AREA* (Stoe & Cie, 2011 ▶); cell refinement: *X-AREA*; data reduction: *X-RED* (Stoe & Cie, 2011 ▶); program(s) used to solve structure: *SIR97* (Altomare *et al.*, 1999 ▶); program(s) used to refine structure: *SHELXL97* (Sheldrick, 2008 ▶); molecular graphics: *PLATON* (Spek, 2009 ▶); software used to prepare material for publication: *PLATON*.

## Supplementary Material

Crystal structure: contains datablock(s) I, New_Global_Publ_Block. DOI: 10.1107/S1600536813030948/bt6943sup1.cif

Structure factors: contains datablock(s) I. DOI: 10.1107/S1600536813030948/bt6943Isup2.hkl

Supplementary material file. DOI: 10.1107/S1600536813030948/bt6943Isup3.cml

Additional supplementary materials:  crystallographic information; 3D view; checkCIF report

## Figures and Tables

**Fig. 1.**
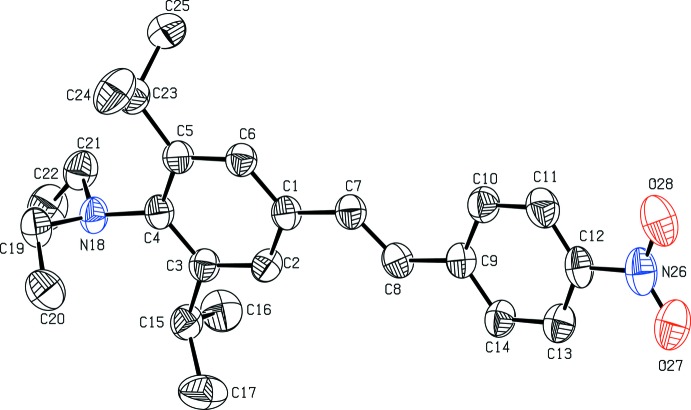
Crystal structure of the title compound with labeling and displacement ellipsoids drawn at the 50% probability level. Second molecule and hydrogen atoms omitted for clearity.

## supplementary crystallographic information

### 1. Comment

The title compound was prepared as a reference compound in a project focusing on chromophores and fluorophores based on quadrupolar donor-acceptor substituted stilbenoid systems, see: Detert & Sugiono (2005); Schmitt *et al.* (2013); and Nemkovich *et al.* (2010). In comparison to the sterically non congested derivative lacking the diisopropyl substitution (Schollmeyer & Detert, 2011), the sterical hindrance around the amino group shifts the UV-vis absorption about 70 nm to the blue and inverts the positive to a negative solvatochromism.

The unit cell is filled with two independent molecules A, B. Parallel layers of molecules A and B are twisted by 79.42 (3)°. As A and B are very similar, only the structural features of A are discussed. The π-system is composed of four almost planar subunits with torsion angles of ±1.6° between nitro group and adjacent phenyl ring. The nitro group is planar (angle sum at N18 = 360°) and the bond length C12—N26 [1.460 (4) Å] is slightly shorter than that of the non-congested analogue, this also holds for the bond lengths of the vinylene group. The torsion angles between the planes of the phenylene rings and the connecting vinylene unit are larger: C6—C1—C7—C8 [175.8 (3)°] and C7—C8—C9—C14 [171.6 (3)°]. Even the vinylene group is twisted: C1—C7—C8—C9 [177.8 (3)°]. Steric congestion around the amino group elongates the aniline C—N bond: 1.434 (3) Å in comparison to 1.385 or 1.378 Å for the compound without 2,6-diisopropyl substitution. Furthermore, the substituents in the 1,2,6-positions adopt an anti,anti conformation with torsion angles of 5.0 (4)° (C15—C3—C4—N18) and 3.7 (4)° (N18—C4—C5—C23). The amino group is pyramidal with an angle sum of 350° on N18. This and the dihedral angles C3—C4—N18—C19 [-69.7 (3)°] and C3—C4—N18—C21 [106.9 (3)°] are structural indicators for an electronic decoupling of the amino group from the acceptor-substituted stilbene unit and therefore the inhibition of the charge transfer and the blue-shifted absorption band in the UV.

### 2. Experimental

The title compound was prepared by adding potassium *tert*-butylate (0.167 g, 1.5 mmol) under nitrogen to a cooled solution of 4-*N*,*N*-diethylamino3,5-diisopropylbenzaldehyde (0.26 g, 0.6 mmol) and diethyl *p*-nitrobenzylphosphonate (0.313 g, 1.1 mmol) in THF (anhyd., 40 ml) and the mixture was stirred for 40 min at 273 K and for further 1 h at ambient temperature. Water (70 ml) was added, the mixture was extracted with chloroform (3 *x* 30 ml) and the pooled organic solutions were washed with brine (3 *x* 20 ml), dried (MgSO_4_), concentrated *in vacuo* and the title compound was isolated in 80% yield from the red oil by chromatography on silica gel using petrouleum ether / ethyl acetate (9 / 1). Yellow needles with with m.p. = 408 - 409 K were obtained by slow evaporation of a solution of the title compound in methanol/chloroform.

### 3. Refinement

Hydrogen atoms were placed at calculated positions with C—H = 0.95 Å (aromatic) or 0.98–0.99 Å (*sp*^3^ C-atoms). All H atoms were refined in the riding-model approximation with isotropic displacement parameters (set at 1.2–1.5 times of the *U*_eq_ of the parent atom).

### Crystal data

**Table:**

C_24_H_32_N_2_O_2_	*Z* = 4
*M**_r_* = 380.52	*F*(000) = 824
Triclinic, *P*1	*D*_x_ = 1.133 Mg m^−^^3^
Hall symbol: -P 1	Melting point: 408 K
*a* = 7.3477 (7) Å	Mo *K*α radiation, λ = 0.71073 Å
*b* = 15.4143 (14) Å	Cell parameters from 12450 reflections
*c* = 20.3747 (19) Å	θ = 2.6–27.5°
α = 104.642 (8)°	µ = 0.07 mm^−^^1^
β = 92.414 (8)°	*T* = 193 K
γ = 90.793 (7)°	Needle, yellow
*V* = 2230.0 (4) Å^3^	0.60 × 0.10 × 0.05 mm

### Data collection

**Table:**

Stoe IPDS 2T diffractometer	3887 reflections with *I* > 2σ(*I*)
Radiation source: sealed X-ray tube, 12 x 0.4 mm long-fine focus	*R*_int_ = 0.086
Plane graphite monochromator	θ_max_ = 28.0°, θ_min_ = 2.7°
Detector resolution: 6.67 pixels mm^-1^	*h* = −9→9
rotation method scans	*k* = −20→19
21656 measured reflections	*l* = 0→26
10640 independent reflections	

### Refinement

**Table:**

Refinement on *F*^2^	Secondary atom site location: difference Fourier map
Least-squares matrix: full	Hydrogen site location: inferred from neighbouring sites
*R*[*F*^2^ > 2σ(*F*^2^)] = 0.057	H-atom parameters constrained
*wR*(*F*^2^) = 0.174	*w* = 1/[σ^2^(*F*_o_^2^) + (0.0886*P*)^2^] where *P* = (*F*_o_^2^ + 2*F*_c_^2^)/3
*S* = 0.79	(Δ/σ)_max_ < 0.001
10640 reflections	Δρ_max_ = 0.25 e Å^−^^3^
518 parameters	Δρ_min_ = −0.24 e Å^−^^3^
0 restraints	Extinction correction: *SHELXL97* (Sheldrick, 2008), Fc^*^=kFc[1+0.001xFc^2^λ^3^/sin(2θ)]^-1/4^
Primary atom site location: structure-invariant direct methods	Extinction coefficient: 0.0087 (11)

### Special details

**Table:**

Experimental. ^1^H-NMR (CDCl_3_): δ = 8.22 ("d", J = 8.8 Hz, 2 H, 3-H, 5-H, Ph—NO_2_); 7.64 ("d", J = 8.8 Hz, 2 H, 2-H, 6-H, Ph—NO_2_); 7.27 (d, J = 16.3 Hz, 1 H, vin); 7.27 (s, 2 H, 3-H, 5-H Ph); 7.08 (d, J = 16.3 Hz, 1 H, vin); 3.51 (sept, J = 6.9 Hz, 2 H, 2-H, i-prop); 3.09 (q, 4 H, NCH_2_); 1.23 (d, J = 6.9 Hz, 12 H,1-H, 3-H i-prop); 1.05 (t, J = 7.1 Hz, 6 C CH_3_ et) 1.27 (m, 12 H, CH_2_); 0.90 ("t", 6 H, CH_3_). ^13^C-NMR(CDCl_3_): δ = 150.4 (C-1, Ph), 146.9 (C-4 PhNO_2_), 146.7 (C-1 PhNO_2_), 144.5 (C-2, C-6 Ph), 134.0 (C-1 vin), 124.3 (C-2, C-6 PhNO_2_), 123.0 (C-3, C-5 PhNO_2_), 49.3 (NCH_2_), 28.0 (CH i-pr), 24.6 (CH_3_ i-pr), 15.4 (CH_3_ et). IR (ATR) 2959, 2931, 2871, 1633, 1590, 1512, 1457, 1336, 1186, 1105, 964, 862, 745, 6890 cm^-1^. HR-ESI-MS:found: 381.2545, calcd for (*M*+H^+^): 381.2542. UV-Vis: λ'max = 364 nm (cyclohexane); λ'max = 347 nm (toluene); λ'max = 344 nm, ε = 29304 l mol-1 cm-1 (dichloromethane); λ'max = 344 nm (acetonitrile).
Geometry. All e.s.d.'s (except the e.s.d. in the dihedral angle between two l.s. planes) are estimated using the full covariance matrix. The cell e.s.d.'s are taken into account individually in the estimation of e.s.d.'s in distances, angles and torsion angles; correlations between e.s.d.'s in cell parameters are only used when they are defined by crystal symmetry. An approximate (isotropic) treatment of cell e.s.d.'s is used for estimating e.s.d.'s involving l.s. planes.
Refinement. Refinement of *F*^2^ against ALL reflections. The weighted *R*-factor *wR* and goodness of fit *S* are based on *F*^2^, conventional *R*-factors *R* are based on *F*, with *F* set to zero for negative *F*^2^. The threshold expression of *F*^2^ > σ(*F*^2^) is used only for calculating *R*-factors(gt) *etc*. and is not relevant to the choice of reflections for refinement. *R*-factors based on *F*^2^ are statistically about twice as large as those based on *F*, and *R*- factors based on ALL data will be even larger.

### Fractional atomic coordinates and isotropic or equivalent isotropic displacement parameters (Å^2^)

**Table:**

	*x*	*y*	*z*	*U*_iso_*/*U*_eq_	
C1	0.4949 (3)	0.30348 (17)	0.27877 (12)	0.0396 (6)	
C2	0.5948 (4)	0.26657 (17)	0.22148 (13)	0.0422 (6)	
H2	0.6339	0.2065	0.2140	0.051*	
C3	0.6378 (3)	0.31579 (17)	0.17551 (13)	0.0410 (6)	
C4	0.5801 (3)	0.40409 (17)	0.18589 (12)	0.0394 (6)	
C5	0.4832 (3)	0.44422 (17)	0.24438 (13)	0.0412 (6)	
C6	0.4431 (4)	0.39198 (17)	0.28856 (13)	0.0422 (6)	
H6	0.3768	0.4181	0.3274	0.051*	
C7	0.4422 (4)	0.25363 (18)	0.32733 (13)	0.0429 (6)	
H7	0.3810	0.2859	0.3658	0.051*	
C8	0.4714 (3)	0.16727 (18)	0.32324 (13)	0.0428 (6)	
H8	0.5366	0.1356	0.2857	0.051*	
C9	0.4133 (3)	0.11634 (17)	0.37066 (13)	0.0403 (6)	
C10	0.3318 (4)	0.15621 (19)	0.43165 (14)	0.0499 (7)	
H10	0.3172	0.2193	0.4443	0.060*	
C11	0.2725 (4)	0.1049 (2)	0.47359 (15)	0.0554 (7)	
H11	0.2174	0.1322	0.5148	0.067*	
C12	0.2945 (4)	0.0136 (2)	0.45466 (14)	0.0475 (7)	
C13	0.3759 (4)	−0.0285 (2)	0.39530 (15)	0.0512 (7)	
H13	0.3913	−0.0915	0.3832	0.061*	
C14	0.4338 (4)	0.02433 (18)	0.35431 (14)	0.0470 (7)	
H14	0.4899	−0.0035	0.3134	0.056*	
C15	0.7519 (4)	0.27354 (18)	0.11555 (13)	0.0469 (6)	
H15	0.7405	0.3114	0.0824	0.056*	
C16	0.9520 (4)	0.2754 (2)	0.13855 (17)	0.0641 (8)	
H16A	0.9907	0.3371	0.1617	0.096*	
H16B	1.0255	0.2536	0.0989	0.096*	
H16C	0.9687	0.2369	0.1698	0.096*	
C17	0.6881 (5)	0.1789 (2)	0.07859 (17)	0.0735 (10)	
H17A	0.7058	0.1391	0.1088	0.110*	
H17B	0.7587	0.1577	0.0381	0.110*	
H17C	0.5586	0.1790	0.0650	0.110*	
N18	0.6186 (3)	0.45126 (14)	0.13554 (11)	0.0433 (5)	
C19	0.4644 (4)	0.4686 (2)	0.09364 (15)	0.0543 (7)	
H19A	0.5098	0.4964	0.0585	0.065*	
H19B	0.3838	0.5117	0.1224	0.065*	
C20	0.3550 (4)	0.3842 (2)	0.05932 (16)	0.0628 (8)	
H20A	0.4335	0.3415	0.0301	0.094*	
H20B	0.2536	0.3992	0.0317	0.094*	
H20C	0.3069	0.3571	0.0939	0.094*	
C21	0.7607 (4)	0.5215 (2)	0.15235 (15)	0.0531 (7)	
H21A	0.8299	0.5176	0.1942	0.064*	
H21B	0.7035	0.5809	0.1614	0.064*	
C22	0.8914 (5)	0.5135 (2)	0.09515 (18)	0.0709 (9)	
H22A	0.9481	0.4548	0.0860	0.106*	
H22B	0.9860	0.5609	0.1085	0.106*	
H22C	0.8240	0.5197	0.0541	0.106*	
C23	0.4226 (4)	0.54056 (18)	0.26072 (14)	0.0485 (7)	
H23	0.4635	0.5677	0.2240	0.058*	
C24	0.2156 (5)	0.5448 (2)	0.2612 (2)	0.0734 (10)	
H24A	0.1723	0.5217	0.2985	0.110*	
H24B	0.1624	0.5084	0.2179	0.110*	
H24C	0.1790	0.6072	0.2675	0.110*	
C25	0.5106 (5)	0.5954 (2)	0.32779 (16)	0.0707 (9)	
H25A	0.4773	0.5685	0.3645	0.106*	
H25B	0.4678	0.6570	0.3371	0.106*	
H25C	0.6434	0.5958	0.3248	0.106*	
N26	0.2300 (3)	−0.0413 (2)	0.49818 (15)	0.0605 (7)	
O27	0.2465 (4)	−0.12263 (18)	0.48017 (13)	0.0808 (7)	
O28	0.1604 (3)	−0.00304 (18)	0.55168 (12)	0.0764 (7)	
C30	−0.0084 (3)	0.05172 (17)	0.27794 (13)	0.0409 (6)	
C31	−0.0659 (4)	−0.03038 (17)	0.28669 (13)	0.0435 (6)	
H31	−0.1336	−0.0312	0.3253	0.052*	
C32	−0.0293 (3)	−0.11158 (17)	0.24173 (13)	0.0412 (6)	
C33	0.0694 (3)	−0.11000 (16)	0.18411 (13)	0.0394 (6)	
C34	0.1346 (3)	−0.02828 (17)	0.17470 (13)	0.0400 (6)	
C35	0.0955 (3)	0.05126 (17)	0.22143 (13)	0.0421 (6)	
H35	0.1400	0.1066	0.2150	0.050*	
C36	−0.0575 (4)	0.13348 (18)	0.32684 (13)	0.0434 (6)	
H36	−0.1172	0.1261	0.3656	0.052*	
C37	−0.0280 (3)	0.21747 (18)	0.32317 (13)	0.0430 (6)	
H37	0.0367	0.2249	0.2855	0.052*	
C38	−0.0844 (3)	0.29959 (18)	0.37077 (13)	0.0419 (6)	
C39	−0.0606 (4)	0.38122 (17)	0.35484 (14)	0.0464 (6)	
H39	−0.0026	0.3826	0.3143	0.056*	
C40	−0.1185 (4)	0.46049 (19)	0.39597 (14)	0.0509 (7)	
H40	−0.1008	0.5158	0.3843	0.061*	
C41	−0.2031 (4)	0.45700 (18)	0.45468 (14)	0.0474 (7)	
C42	−0.2282 (4)	0.3779 (2)	0.47287 (15)	0.0563 (8)	
H42	−0.2856	0.3774	0.5137	0.068*	
C43	−0.1692 (4)	0.29890 (19)	0.43136 (14)	0.0513 (7)	
H43	−0.1859	0.2440	0.4437	0.062*	
C44	−0.0981 (4)	−0.19792 (18)	0.25670 (15)	0.0494 (7)	
H44	−0.0614	−0.2491	0.2191	0.059*	
C45	−0.3050 (4)	−0.2008 (2)	0.2578 (2)	0.0739 (10)	
H45A	−0.3464	−0.2596	0.2623	0.111*	
H45B	−0.3574	−0.1906	0.2154	0.111*	
H45C	−0.3445	−0.1539	0.2963	0.111*	
C46	−0.0117 (5)	−0.2105 (2)	0.32264 (17)	0.0732 (10)	
H46A	−0.0399	−0.1594	0.3601	0.110*	
H46B	0.1206	−0.2143	0.3190	0.110*	
H46C	−0.0603	−0.2659	0.3313	0.110*	
N47	0.1061 (3)	−0.19036 (14)	0.13296 (11)	0.0426 (5)	
C48	0.2394 (4)	−0.25017 (19)	0.15094 (15)	0.0494 (7)	
H48A	0.1751	−0.3007	0.1627	0.059*	
H48B	0.3134	−0.2173	0.1916	0.059*	
C49	0.3639 (5)	−0.2868 (2)	0.09384 (18)	0.0682 (9)	
H49A	0.2937	−0.3266	0.0558	0.102*	
H49B	0.4609	−0.3204	0.1099	0.102*	
H49C	0.4178	−0.2370	0.0788	0.102*	
C50	−0.0487 (4)	−0.23375 (19)	0.08914 (15)	0.0534 (7)	
H50A	−0.1341	−0.2586	0.1164	0.064*	
H50B	−0.0048	−0.2844	0.0535	0.064*	
C51	−0.1488 (4)	−0.1704 (2)	0.05593 (16)	0.0600 (8)	
H51A	−0.1947	−0.1208	0.0910	0.090*	
H51B	−0.2512	−0.2026	0.0272	0.090*	
H51C	−0.0656	−0.1467	0.0280	0.090*	
C52	0.2523 (4)	−0.02585 (18)	0.11595 (13)	0.0454 (6)	
H52	0.2337	−0.0848	0.0816	0.054*	
C53	0.4520 (4)	−0.0174 (2)	0.13822 (17)	0.0643 (8)	
H53A	0.4758	0.0401	0.1717	0.096*	
H53B	0.5256	−0.0202	0.0988	0.096*	
H53C	0.4841	−0.0665	0.1587	0.096*	
C54	0.2021 (5)	0.0470 (2)	0.08046 (17)	0.0675 (9)	
H54A	0.0725	0.0407	0.0663	0.101*	
H54B	0.2751	0.0410	0.0405	0.101*	
H54C	0.2264	0.1060	0.1118	0.101*	
N55	−0.2667 (4)	0.54027 (19)	0.49826 (14)	0.0589 (7)	
O56	−0.3403 (3)	0.53623 (16)	0.55103 (12)	0.0761 (7)	
O57	−0.2477 (4)	0.60986 (16)	0.48113 (13)	0.0790 (7)	

### Atomic displacement parameters (Å^2^)

**Table:**

	*U*^11^	*U*^22^	*U*^33^	*U*^12^	*U*^13^	*U*^23^
C1	0.0364 (13)	0.0449 (15)	0.0405 (13)	−0.0010 (11)	0.0036 (11)	0.0161 (11)
C2	0.0424 (14)	0.0391 (14)	0.0476 (15)	0.0048 (11)	0.0065 (12)	0.0150 (12)
C3	0.0399 (14)	0.0428 (15)	0.0423 (14)	0.0019 (11)	0.0037 (11)	0.0140 (12)
C4	0.0372 (13)	0.0448 (14)	0.0400 (13)	−0.0021 (11)	0.0037 (11)	0.0174 (11)
C5	0.0395 (14)	0.0431 (14)	0.0419 (14)	0.0014 (11)	0.0013 (11)	0.0125 (11)
C6	0.0421 (14)	0.0438 (15)	0.0423 (14)	0.0017 (11)	0.0039 (11)	0.0135 (12)
C7	0.0410 (14)	0.0481 (16)	0.0425 (14)	0.0015 (11)	0.0051 (11)	0.0164 (12)
C8	0.0386 (14)	0.0522 (17)	0.0401 (13)	−0.0007 (12)	0.0060 (11)	0.0160 (12)
C9	0.0339 (13)	0.0478 (15)	0.0428 (14)	0.0005 (11)	0.0035 (11)	0.0179 (12)
C10	0.0558 (17)	0.0504 (16)	0.0474 (16)	0.0062 (13)	0.0077 (13)	0.0186 (13)
C11	0.0573 (18)	0.068 (2)	0.0470 (16)	0.0048 (15)	0.0106 (14)	0.0234 (15)
C12	0.0405 (15)	0.0644 (19)	0.0469 (15)	−0.0022 (13)	0.0023 (12)	0.0313 (14)
C13	0.0504 (17)	0.0525 (17)	0.0565 (17)	0.0022 (13)	0.0057 (14)	0.0238 (14)
C14	0.0472 (16)	0.0493 (16)	0.0504 (16)	0.0068 (13)	0.0090 (13)	0.0222 (13)
C15	0.0517 (16)	0.0474 (16)	0.0460 (15)	0.0019 (13)	0.0136 (13)	0.0181 (12)
C16	0.0511 (18)	0.077 (2)	0.0655 (19)	0.0110 (16)	0.0166 (15)	0.0184 (17)
C17	0.088 (3)	0.062 (2)	0.063 (2)	−0.0143 (18)	0.0276 (19)	−0.0017 (16)
N18	0.0454 (13)	0.0452 (12)	0.0456 (12)	−0.0052 (10)	0.0013 (10)	0.0233 (10)
C19	0.0585 (18)	0.0606 (18)	0.0492 (16)	0.0001 (14)	−0.0021 (14)	0.0251 (14)
C20	0.0538 (18)	0.076 (2)	0.0578 (18)	−0.0047 (16)	−0.0032 (15)	0.0163 (16)
C21	0.0496 (16)	0.0509 (17)	0.0635 (18)	−0.0066 (13)	0.0020 (14)	0.0237 (14)
C22	0.067 (2)	0.073 (2)	0.083 (2)	−0.0051 (17)	0.0207 (18)	0.0356 (19)
C23	0.0504 (16)	0.0475 (16)	0.0509 (16)	0.0029 (13)	0.0074 (13)	0.0178 (13)
C24	0.061 (2)	0.061 (2)	0.106 (3)	0.0192 (17)	0.0203 (19)	0.032 (2)
C25	0.103 (3)	0.0473 (18)	0.0601 (19)	−0.0032 (18)	0.0006 (19)	0.0115 (15)
N26	0.0478 (15)	0.081 (2)	0.0655 (17)	−0.0073 (13)	−0.0017 (13)	0.0430 (16)
O27	0.0948 (19)	0.0746 (17)	0.0894 (18)	−0.0072 (14)	0.0134 (15)	0.0504 (14)
O28	0.0732 (16)	0.110 (2)	0.0594 (14)	−0.0038 (14)	0.0148 (12)	0.0438 (14)
C30	0.0378 (14)	0.0434 (15)	0.0426 (14)	−0.0004 (11)	0.0001 (11)	0.0133 (12)
C31	0.0418 (14)	0.0456 (15)	0.0462 (15)	0.0020 (12)	0.0076 (12)	0.0166 (12)
C32	0.0387 (14)	0.0416 (15)	0.0467 (14)	0.0006 (11)	0.0051 (11)	0.0172 (12)
C33	0.0390 (14)	0.0374 (14)	0.0419 (14)	0.0012 (11)	0.0042 (11)	0.0097 (11)
C34	0.0373 (14)	0.0423 (15)	0.0410 (14)	0.0009 (11)	0.0021 (11)	0.0119 (11)
C35	0.0405 (14)	0.0417 (15)	0.0468 (15)	−0.0015 (11)	0.0031 (12)	0.0162 (12)
C36	0.0415 (14)	0.0464 (16)	0.0428 (14)	0.0024 (12)	0.0048 (11)	0.0117 (12)
C37	0.0394 (14)	0.0477 (16)	0.0422 (14)	0.0038 (12)	0.0027 (11)	0.0113 (12)
C38	0.0353 (13)	0.0469 (15)	0.0427 (14)	0.0005 (11)	−0.0007 (11)	0.0105 (12)
C39	0.0483 (16)	0.0409 (15)	0.0484 (15)	0.0030 (12)	0.0057 (13)	0.0079 (12)
C40	0.0507 (17)	0.0450 (16)	0.0543 (17)	0.0005 (13)	0.0041 (14)	0.0074 (13)
C41	0.0443 (15)	0.0466 (16)	0.0439 (15)	0.0055 (12)	0.0016 (12)	−0.0023 (12)
C42	0.0598 (19)	0.0605 (19)	0.0441 (16)	0.0028 (15)	0.0105 (14)	0.0036 (14)
C43	0.0587 (18)	0.0492 (17)	0.0461 (15)	−0.0032 (14)	0.0076 (13)	0.0119 (13)
C44	0.0496 (16)	0.0440 (16)	0.0580 (17)	−0.0004 (12)	0.0095 (13)	0.0182 (13)
C45	0.0555 (19)	0.063 (2)	0.111 (3)	−0.0070 (16)	0.0199 (19)	0.034 (2)
C46	0.101 (3)	0.059 (2)	0.067 (2)	0.0040 (19)	0.001 (2)	0.0289 (17)
N47	0.0440 (12)	0.0370 (12)	0.0449 (12)	0.0012 (9)	−0.0009 (10)	0.0076 (9)
C48	0.0472 (16)	0.0462 (16)	0.0569 (17)	0.0052 (13)	0.0036 (13)	0.0166 (13)
C49	0.067 (2)	0.061 (2)	0.079 (2)	0.0191 (17)	0.0183 (18)	0.0189 (17)
C50	0.0536 (17)	0.0487 (16)	0.0535 (16)	−0.0061 (13)	−0.0026 (13)	0.0060 (13)
C51	0.0525 (18)	0.064 (2)	0.0614 (18)	0.0012 (15)	−0.0079 (15)	0.0133 (15)
C52	0.0513 (16)	0.0414 (15)	0.0449 (14)	0.0012 (12)	0.0120 (12)	0.0122 (12)
C53	0.0518 (18)	0.078 (2)	0.070 (2)	0.0060 (16)	0.0184 (16)	0.0281 (17)
C54	0.074 (2)	0.079 (2)	0.0608 (19)	0.0123 (18)	0.0169 (17)	0.0364 (17)
N55	0.0510 (15)	0.0601 (18)	0.0558 (16)	0.0056 (13)	−0.0056 (12)	−0.0026 (13)
O56	0.0750 (16)	0.0849 (17)	0.0544 (13)	0.0091 (13)	0.0146 (12)	−0.0105 (12)
O57	0.0921 (18)	0.0523 (14)	0.0835 (17)	0.0146 (13)	0.0113 (14)	−0.0014 (13)

### Geometric parameters (Å, º)

**Table:**

C1—C6	1.389 (4)	C30—C31	1.385 (4)
C1—C2	1.405 (3)	C30—C35	1.406 (4)
C1—C7	1.458 (3)	C30—C36	1.456 (4)
C2—C3	1.389 (4)	C31—C32	1.389 (4)
C2—H2	0.9500	C31—H31	0.9500
C3—C4	1.398 (3)	C32—C33	1.411 (3)
C3—C15	1.523 (4)	C32—C44	1.523 (4)
C4—C5	1.421 (3)	C33—C34	1.402 (3)
C4—N18	1.434 (3)	C33—N47	1.440 (3)
C5—C6	1.388 (4)	C34—C35	1.391 (4)
C5—C23	1.515 (4)	C34—C52	1.514 (3)
C6—H6	0.9500	C35—H35	0.9500
C7—C8	1.333 (4)	C36—C37	1.331 (4)
C7—H7	0.9500	C36—H36	0.9500
C8—C9	1.463 (3)	C37—C38	1.464 (4)
C8—H8	0.9500	C37—H37	0.9500
C9—C14	1.384 (4)	C38—C39	1.387 (4)
C9—C10	1.402 (4)	C38—C43	1.408 (4)
C10—C11	1.382 (4)	C39—C40	1.380 (4)
C10—H10	0.9500	C39—H39	0.9500
C11—C12	1.375 (4)	C40—C41	1.384 (4)
C11—H11	0.9500	C40—H40	0.9500
C12—C13	1.384 (4)	C41—C42	1.374 (4)
C12—N26	1.460 (4)	C41—N55	1.459 (4)
C13—C14	1.380 (4)	C42—C43	1.382 (4)
C13—H13	0.9500	C42—H42	0.9500
C14—H14	0.9500	C43—H43	0.9500
C15—C17	1.520 (4)	C44—C46	1.519 (4)
C15—C16	1.522 (4)	C44—C45	1.522 (4)
C15—H15	1.0000	C44—H44	1.0000
C16—H16A	0.9800	C45—H45A	0.9800
C16—H16B	0.9800	C45—H45B	0.9800
C16—H16C	0.9800	C45—H45C	0.9800
C17—H17A	0.9800	C46—H46A	0.9800
C17—H17B	0.9800	C46—H46B	0.9800
C17—H17C	0.9800	C46—H46C	0.9800
N18—C19	1.458 (3)	N47—C48	1.452 (3)
N18—C21	1.460 (3)	N47—C50	1.459 (3)
C19—C20	1.514 (4)	C48—C49	1.510 (4)
C19—H19A	0.9900	C48—H48A	0.9900
C19—H19B	0.9900	C48—H48B	0.9900
C20—H20A	0.9800	C49—H49A	0.9800
C20—H20B	0.9800	C49—H49B	0.9800
C20—H20C	0.9800	C49—H49C	0.9800
C21—C22	1.523 (4)	C50—C51	1.504 (4)
C21—H21A	0.9900	C50—H50A	0.9900
C21—H21B	0.9900	C50—H50B	0.9900
C22—H22A	0.9800	C51—H51A	0.9800
C22—H22B	0.9800	C51—H51B	0.9800
C22—H22C	0.9800	C51—H51C	0.9800
C23—C24	1.523 (4)	C52—C53	1.511 (4)
C23—C25	1.525 (4)	C52—C54	1.522 (4)
C23—H23	1.0000	C52—H52	1.0000
C24—H24A	0.9800	C53—H53A	0.9800
C24—H24B	0.9800	C53—H53B	0.9800
C24—H24C	0.9800	C53—H53C	0.9800
C25—H25A	0.9800	C54—H54A	0.9800
C25—H25B	0.9800	C54—H54B	0.9800
C25—H25C	0.9800	C54—H54C	0.9800
N26—O27	1.223 (3)	N55—O57	1.217 (3)
N26—O28	1.236 (3)	N55—O56	1.239 (3)
			
C6—C1—C2	117.4 (2)	C31—C30—C35	117.5 (2)
C6—C1—C7	119.1 (2)	C31—C30—C36	119.1 (2)
C2—C1—C7	123.5 (2)	C35—C30—C36	123.4 (2)
C3—C2—C1	121.6 (2)	C30—C31—C32	123.1 (2)
C3—C2—H2	119.2	C30—C31—H31	118.5
C1—C2—H2	119.2	C32—C31—H31	118.5
C2—C3—C4	119.7 (2)	C31—C32—C33	118.3 (2)
C2—C3—C15	119.4 (2)	C31—C32—C44	118.6 (2)
C4—C3—C15	120.9 (2)	C33—C32—C44	123.1 (2)
C3—C4—C5	120.1 (2)	C34—C33—C32	120.2 (2)
C3—C4—N18	118.1 (2)	C34—C33—N47	117.5 (2)
C5—C4—N18	121.8 (2)	C32—C33—N47	122.3 (2)
C6—C5—C4	117.9 (2)	C35—C34—C33	119.4 (2)
C6—C5—C23	118.8 (2)	C35—C34—C52	119.7 (2)
C4—C5—C23	123.3 (2)	C33—C34—C52	120.9 (2)
C5—C6—C1	123.3 (2)	C34—C35—C30	121.5 (2)
C5—C6—H6	118.4	C34—C35—H35	119.2
C1—C6—H6	118.4	C30—C35—H35	119.2
C8—C7—C1	126.6 (2)	C37—C36—C30	127.1 (2)
C8—C7—H7	116.7	C37—C36—H36	116.4
C1—C7—H7	116.7	C30—C36—H36	116.4
C7—C8—C9	126.7 (2)	C36—C37—C38	127.2 (2)
C7—C8—H8	116.6	C36—C37—H37	116.4
C9—C8—H8	116.6	C38—C37—H37	116.4
C14—C9—C10	117.9 (2)	C39—C38—C43	118.2 (2)
C14—C9—C8	119.2 (2)	C39—C38—C37	119.3 (2)
C10—C9—C8	122.9 (2)	C43—C38—C37	122.5 (2)
C11—C10—C9	120.8 (3)	C40—C39—C38	122.1 (3)
C11—C10—H10	119.6	C40—C39—H39	118.9
C9—C10—H10	119.6	C38—C39—H39	118.9
C12—C11—C10	119.0 (3)	C39—C40—C41	118.0 (3)
C12—C11—H11	120.5	C39—C40—H40	121.0
C10—C11—H11	120.5	C41—C40—H40	121.0
C11—C12—C13	122.2 (2)	C42—C41—C40	121.9 (2)
C11—C12—N26	119.4 (3)	C42—C41—N55	119.5 (3)
C13—C12—N26	118.5 (3)	C40—C41—N55	118.5 (3)
C14—C13—C12	117.7 (3)	C41—C42—C43	119.5 (3)
C14—C13—H13	121.2	C41—C42—H42	120.2
C12—C13—H13	121.2	C43—C42—H42	120.2
C13—C14—C9	122.4 (3)	C42—C43—C38	120.2 (3)
C13—C14—H14	118.8	C42—C43—H43	119.9
C9—C14—H14	118.8	C38—C43—H43	119.9
C17—C15—C16	110.8 (3)	C46—C44—C45	111.0 (3)
C17—C15—C3	113.3 (2)	C46—C44—C32	111.4 (2)
C16—C15—C3	110.2 (2)	C45—C44—C32	111.2 (2)
C17—C15—H15	107.4	C46—C44—H44	107.7
C16—C15—H15	107.4	C45—C44—H44	107.7
C3—C15—H15	107.4	C32—C44—H44	107.7
C15—C16—H16A	109.5	C44—C45—H45A	109.5
C15—C16—H16B	109.5	C44—C45—H45B	109.5
H16A—C16—H16B	109.5	H45A—C45—H45B	109.5
C15—C16—H16C	109.5	C44—C45—H45C	109.5
H16A—C16—H16C	109.5	H45A—C45—H45C	109.5
H16B—C16—H16C	109.5	H45B—C45—H45C	109.5
C15—C17—H17A	109.5	C44—C46—H46A	109.5
C15—C17—H17B	109.5	C44—C46—H46B	109.5
H17A—C17—H17B	109.5	H46A—C46—H46B	109.5
C15—C17—H17C	109.5	C44—C46—H46C	109.5
H17A—C17—H17C	109.5	H46A—C46—H46C	109.5
H17B—C17—H17C	109.5	H46B—C46—H46C	109.5
C4—N18—C19	116.9 (2)	C33—N47—C48	117.2 (2)
C4—N18—C21	117.8 (2)	C33—N47—C50	116.5 (2)
C19—N18—C21	115.3 (2)	C48—N47—C50	115.8 (2)
N18—C19—C20	112.6 (2)	N47—C48—C49	112.1 (2)
N18—C19—H19A	109.1	N47—C48—H48A	109.2
C20—C19—H19A	109.1	C49—C48—H48A	109.2
N18—C19—H19B	109.1	N47—C48—H48B	109.2
C20—C19—H19B	109.1	C49—C48—H48B	109.2
H19A—C19—H19B	107.8	H48A—C48—H48B	107.9
C19—C20—H20A	109.5	C48—C49—H49A	109.5
C19—C20—H20B	109.5	C48—C49—H49B	109.5
H20A—C20—H20B	109.5	H49A—C49—H49B	109.5
C19—C20—H20C	109.5	C48—C49—H49C	109.5
H20A—C20—H20C	109.5	H49A—C49—H49C	109.5
H20B—C20—H20C	109.5	H49B—C49—H49C	109.5
N18—C21—C22	111.7 (3)	N47—C50—C51	112.6 (2)
N18—C21—H21A	109.3	N47—C50—H50A	109.1
C22—C21—H21A	109.3	C51—C50—H50A	109.1
N18—C21—H21B	109.3	N47—C50—H50B	109.1
C22—C21—H21B	109.3	C51—C50—H50B	109.1
H21A—C21—H21B	107.9	H50A—C50—H50B	107.8
C21—C22—H22A	109.5	C50—C51—H51A	109.5
C21—C22—H22B	109.5	C50—C51—H51B	109.5
H22A—C22—H22B	109.5	H51A—C51—H51B	109.5
C21—C22—H22C	109.5	C50—C51—H51C	109.5
H22A—C22—H22C	109.5	H51A—C51—H51C	109.5
H22B—C22—H22C	109.5	H51B—C51—H51C	109.5
C5—C23—C24	110.9 (2)	C53—C52—C34	111.0 (2)
C5—C23—C25	111.5 (2)	C53—C52—C54	110.3 (3)
C24—C23—C25	111.1 (3)	C34—C52—C54	113.8 (2)
C5—C23—H23	107.7	C53—C52—H52	107.1
C24—C23—H23	107.7	C34—C52—H52	107.1
C25—C23—H23	107.7	C54—C52—H52	107.1
C23—C24—H24A	109.5	C52—C53—H53A	109.5
C23—C24—H24B	109.5	C52—C53—H53B	109.5
H24A—C24—H24B	109.5	H53A—C53—H53B	109.5
C23—C24—H24C	109.5	C52—C53—H53C	109.5
H24A—C24—H24C	109.5	H53A—C53—H53C	109.5
H24B—C24—H24C	109.5	H53B—C53—H53C	109.5
C23—C25—H25A	109.5	C52—C54—H54A	109.5
C23—C25—H25B	109.5	C52—C54—H54B	109.5
H25A—C25—H25B	109.5	H54A—C54—H54B	109.5
C23—C25—H25C	109.5	C52—C54—H54C	109.5
H25A—C25—H25C	109.5	H54A—C54—H54C	109.5
H25B—C25—H25C	109.5	H54B—C54—H54C	109.5
O27—N26—O28	123.1 (3)	O57—N55—O56	123.0 (3)
O27—N26—C12	118.9 (3)	O57—N55—C41	119.2 (3)
O28—N26—C12	118.0 (3)	O56—N55—C41	117.8 (3)
			
C6—C1—C2—C3	−1.1 (4)	C35—C30—C31—C32	−1.5 (4)
C7—C1—C2—C3	178.2 (3)	C36—C30—C31—C32	178.4 (3)
C1—C2—C3—C4	−0.3 (4)	C30—C31—C32—C33	−0.8 (4)
C1—C2—C3—C15	177.9 (2)	C30—C31—C32—C44	179.5 (2)
C2—C3—C4—C5	2.1 (4)	C31—C32—C33—C34	2.7 (4)
C15—C3—C4—C5	−176.1 (2)	C44—C32—C33—C34	−177.6 (2)
C2—C3—C4—N18	−176.8 (2)	C31—C32—C33—N47	−176.9 (2)
C15—C3—C4—N18	5.0 (4)	C44—C32—C33—N47	2.8 (4)
C3—C4—C5—C6	−2.3 (4)	C32—C33—C34—C35	−2.2 (4)
N18—C4—C5—C6	176.5 (2)	N47—C33—C34—C35	177.3 (2)
C3—C4—C5—C23	177.5 (2)	C32—C33—C34—C52	175.5 (2)
N18—C4—C5—C23	−3.7 (4)	N47—C33—C34—C52	−4.9 (4)
C4—C5—C6—C1	0.9 (4)	C33—C34—C35—C30	−0.1 (4)
C23—C5—C6—C1	−179.0 (2)	C52—C34—C35—C30	−177.9 (2)
C2—C1—C6—C5	0.8 (4)	C31—C30—C35—C34	2.0 (4)
C7—C1—C6—C5	−178.5 (3)	C36—C30—C35—C34	−177.9 (2)
C6—C1—C7—C8	175.8 (3)	C31—C30—C36—C37	−173.6 (3)
C2—C1—C7—C8	−3.6 (4)	C35—C30—C36—C37	6.3 (4)
C1—C7—C8—C9	−177.8 (3)	C30—C36—C37—C38	177.1 (3)
C7—C8—C9—C14	171.6 (3)	C36—C37—C38—C39	−172.5 (3)
C7—C8—C9—C10	−6.4 (4)	C36—C37—C38—C43	4.8 (4)
C14—C9—C10—C11	−0.6 (4)	C43—C38—C39—C40	−0.5 (4)
C8—C9—C10—C11	177.4 (3)	C37—C38—C39—C40	176.9 (2)
C9—C10—C11—C12	0.0 (4)	C38—C39—C40—C41	−0.2 (4)
C10—C11—C12—C13	0.7 (4)	C39—C40—C41—C42	0.8 (4)
C10—C11—C12—N26	−179.2 (3)	C39—C40—C41—N55	−179.4 (2)
C11—C12—C13—C14	−0.7 (4)	C40—C41—C42—C43	−0.6 (5)
N26—C12—C13—C14	179.1 (3)	N55—C41—C42—C43	179.5 (3)
C12—C13—C14—C9	0.0 (4)	C41—C42—C43—C38	−0.1 (4)
C10—C9—C14—C13	0.6 (4)	C39—C38—C43—C42	0.7 (4)
C8—C9—C14—C13	−177.5 (3)	C37—C38—C43—C42	−176.7 (3)
C2—C3—C15—C17	46.7 (4)	C31—C32—C44—C46	−63.0 (3)
C4—C3—C15—C17	−135.1 (3)	C33—C32—C44—C46	117.4 (3)
C2—C3—C15—C16	−78.1 (3)	C31—C32—C44—C45	61.4 (3)
C4—C3—C15—C16	100.1 (3)	C33—C32—C44—C45	−118.3 (3)
C3—C4—N18—C19	109.1 (3)	C34—C33—N47—C48	108.3 (3)
C5—C4—N18—C19	−69.7 (3)	C32—C33—N47—C48	−72.1 (3)
C3—C4—N18—C21	−106.9 (3)	C34—C33—N47—C50	−108.3 (3)
C5—C4—N18—C21	74.3 (3)	C32—C33—N47—C50	71.3 (3)
C4—N18—C19—C20	−53.8 (3)	C33—N47—C48—C49	−138.5 (3)
C21—N18—C19—C20	161.4 (2)	C50—N47—C48—C49	77.8 (3)
C4—N18—C21—C22	133.2 (3)	C33—N47—C50—C51	53.3 (3)
C19—N18—C21—C22	−82.3 (3)	C48—N47—C50—C51	−162.7 (2)
C6—C5—C23—C24	−62.7 (3)	C35—C34—C52—C53	80.0 (3)
C4—C5—C23—C24	117.5 (3)	C33—C34—C52—C53	−97.7 (3)
C6—C5—C23—C25	61.6 (3)	C35—C34—C52—C54	−45.1 (4)
C4—C5—C23—C25	−118.2 (3)	C33—C34—C52—C54	137.1 (3)
C11—C12—N26—O27	178.4 (3)	C42—C41—N55—O57	−178.4 (3)
C13—C12—N26—O27	−1.5 (4)	C40—C41—N55—O57	1.7 (4)
C11—C12—N26—O28	−1.4 (4)	C42—C41—N55—O56	0.7 (4)
C13—C12—N26—O28	178.8 (3)	C40—C41—N55—O56	−179.2 (3)
